# NAMPT overexpression alleviates alcohol-induced hepatic steatosis in mice

**DOI:** 10.1371/journal.pone.0212523

**Published:** 2019-02-22

**Authors:** Xiwen Xiong, Jiahui Yu, Rui Fan, Cuicui Zhang, Lin Xu, Xupeng Sun, Yanmei Huang, Qingzhi Wang, Hai-Bin Ruan, Xinlai Qian

**Affiliations:** 1 School of Forensic Medicine, Xinxiang Medical University, Xinxiang, Henan, China; 2 Xinxiang Key Laboratory of Metabolism and Integrative Physiology, Xinxiang Medical University, Xinxiang, Henan, China; 3 School of Basic Medical Sciences, Xinxiang Medical University, Xinxiang, Henan, China; 4 Department of Medical Genetics, School of Basic Medicine, Tongji Medical College, Huazhong University of Science and Technology, Wuhan, Hubei, China; 5 Department of Integrative Biology and Physiology, University of Minnesota Medical School, Minneapolis, MN, United States of America; Macquarie University, AUSTRALIA

## Abstract

Nicotinamide phosphoribosyltransferase (NAMPT) is a rate-limiting enzyme in mammalian nicotinamide adenine dinucleotide (NAD)^+^ biosynthesis. Through its NAD^+^-biosynthetic activity, NAMPT influences the activity of NAD^+^-dependent enzymes, such as sirtuins. NAMPT is able to modulate processes involved in the pathogenesis of non-alcohol induced fatty liver disease (NAFLD), but the roles NAMPT plays in development of alcoholic liver disease (ALD) still remain unknown. Here, we show that ethanol treatment suppresses the expression of *Nampt* in hepatocytes. Consistently, chronic ethanol administration also reduces *Nampt* expression in the mouse liver. We next demonstrate that hepatocytes infected with Ad-NAMPT adenovirus exhibit significantly elevated intracellular NAD^+^ levels and decreased ethanol-induced triglyceride (TG) accumulation. Similarly, adenovirus-mediated overexpression of NAMPT in mice ameliorates ethanol induced hepatic steatosis. Moreover, we demonstrate that SIRT1 is required to mediate the effects of NAMPT on reduction of hepatic TG accumulation and serum ALT, AST levels in ethanol-fed mice. Our results provide important insights in targeting NAMPT for treating alcoholic fatty liver disease.

## Introduction

Excessive alcohol consumption is a global healthcare problem[1]. The liver sustains the greatest degree of tissue injury by alcohol drinking because it is the major organ for alcohol metabolism[2, 3]. Alcoholic liver disease (ALD) is a wide spectrum of clinical liver disorders ranging from hepatic steatosis to other server forms of liver injury, including alcoholic hepatitis, cirrhosis, and superimposed hepatocellular carcinoma. Hepatic steatosis is an early and reversible stage of ALD[4, 5]. More than 90% of heavy drinkers develop steatosis while about 20%-40% of heavy drinkers develop more severe forms of liver injuries such hepatitis and cirrhosis[3, 5]. Hepatic steatosis is characterized by the deposition of fat, such as triglycerides, phospholipids, and cholesterol esters, in hepatocytes[4, 5]. To date, the pathogenesis of alcohol induced hepatic steatosis have not been completely elucidated. Possible underling mechanisms may include enhanced lipogenesis, increased oxidative stress, diminished fatty acids β-oxidation, and impaired VLDL secretion[2–4].

Alcohol is a polar molecule that diffuses easily across the cell membranes. Approximately 90% of ingested alcohol is metabolized in the liver[2]. Alcohol is mainly oxidized to acetaldehyde by alcohol dehydrogenase (ADH) in the cytosol of hepatocytes. Other enzymes, including P450 2E1 (CYP2E1) and catalase, which are respectively present in the microsomes and peroxisomes, also contribute to alcohol oxidation in liver[2, 4, 6]. Then, acetaldehyde dehydrogenase (ALDH) metabolize acetaldehyde to acetate primarily in the mitochondria of hepatocyte. Both ADH and ALDH use nicotinamide adenine dinucleotide (NAD^+^) as a co-factor, producing its reduced form (NADH) in both steps. Therefore, the alcohol metabolism leads to NADH accumulation, causing a consequent reduction of the NAD^+^/NADH ratio. This reduction may affect a lot of metabolism related biochemical reactions, such as glycolysis, the tricarboxylic acid (TCA) cycle and β-oxidation of fatty acids, thereby dysregulating energy metabolism, which contributes to the pathogenesis of alcoholic fatty liver[2, 4, 6]. Thus, restoring the NAD^+^/NADH ratio by upregulating NAD^+^ production may be a good way to ameliorate ethanol-induced hepatic steatosis.

NAD^+^ biosynthesis is accomplished through either the de novo pathway from tryptophan or salvage pathway from three NAD^+^ precursors, nicotinamide (NAM), nicotinic acid (NA) and nicotinamide riboside (NR)[7–9]. The majority of NAD^+^ is synthesized from NAM through the NAD^+^ salvage pathway in mammalian cells. Nicotinamide phosphoribosyltransferase (NAMPT) is the rate-limiting enzyme in the NAD^+^ salvage pathway converting NAM to the intermediate nicotinamide mononucleotide (NMN), which is further converted to NAD^+^ by NMN adenylyltransferases (NMNATs)[10, 11]. Through its ability to produce NAD^+^, NAMPT influences the activity of NAD^+^-dependent enzymes, such as sirtuins and poly(ADP-ribose) polymerases, and further regulates cellular metabolism[10, 12]. Moreover, NAMPT is able to regulate cellular processes involved in the pathogenesis of metabolic disorders, including the oxidative stress response, apoptosis, lipid and glucose metabolism, inflammation and insulin resistance[10–13]. Recently, several studies have demonstrated decreased NAD^+^ levels and/or NAMPT abundance in both animal models and patients with non-alcoholic fatty liver disease (NAFLD)[14–16]. In mouse models of NAFLD, inhibition of NAMPT has been shown to aggravate the development of NAFLD through reducing SIRT1 activity[17–19].

ALD shares similar histopathological and molecular biological features with NAFLD, but the role NAMPT plays in ALD is still unknown[20]. In this study, we show that NAMPT expression is significantly decreased after ethanol treatment in primary hepatocytes or in mouse livers, which is consistent with the reduction of intracellular NAD^+^ levels. Notably, overexpression of NAMPT in hepatocytes upregulates NAD^+^ levels thereby ameliorating ethanol-induced triglyceride accumulation in cells. In a chronic and binge ethanol feeding mouse model, adenovirus-mediated NAMPT transduction in liver cells significantly protects against ethanol-induced hepatic steatosis and injury. In addition, we observed that the protective effects of NAMPT could be abolished by SIRT1 knockdown. Therefore, these findings support a crucial role of NAMPT and NAD^+^ in ALD.

## Materials and methods

### Mice

C57BL/6J mice and Albumin-Cre mouse strain were purchased from Nanjing Biomedical Research Institute of Nanjing University. *Sirt6* floxed mouse strain was provided by Dr. X. Charlie Dong in Indiana University School of Medicine. Mice were maintained in an environmentally-controlled room and fed a rodent chow with free access to water. All animal procedures were performed in accordance with Henan Province Laboratory Animal Care Guidelines for the use of animals in research and were approved by the Institutional Animal Use and Care Committee Xinxiang Medical University.

### Chronic plus binge ethanol feeding

A mouse model of chronic plus binge ethanol consumption was generated as previously described[21]. Briefly, 10-12-week-old of C57BL/6J male mice were fed a liquid control diet (Bio-Serv) for 5 days to give them time to adapt to it. Then, mice were divided into 2 groups: ethanol group mice were fed a liquid diet containing 5% ethanol for 10 days; control group mice were pair-fed an isocaloric control diet for 10 days. On the early morning of day 11 (AM 7:00–8:00), mice in either the ethanol group or control group were oral gavaged with a single dose of ethanol (5g/kg body weight, 31.5% ethanol) or isocaloric dextrin maltose, respectively. The mice were euthanized 9 hours after gavage, and blood and liver tissues were collected.

### Adenovirus preparation

Adenoviruses carrying NAMPT, GFP were generated using the pAdEasy system (Agilent); *Sirt1* shRNA and control shRNA were generated using the BLOCK-iT system (Invitrogen). Adenoviruses were amplified in HEK293A cells and purified by CsCl gradient centrifugation. The viruses were titered using an Adeno-X^TM^ Rapid Titer kit (Takara) according to the manufacturer’s manual.

### Administration of mice with adenovirus

Adenovirus administration was performed on the last day of the acclimatization stage to the liquid diet feeding, then followed by 10 days of ethanol feeding plus one binge. For single adenovirus treatments, mice were injected with 1X10^9^ plaque-forming units (pfu) of adenoviruses through the tail vein. For combination adenovirus treatments, four groups of mice were injected with either Ad-GFP and control shRNA, Ad-NAMPT and control shRNA, Ad-GFP and *Sirt1* shRNA, or Ad-NAMPT and *Sirt1* shRNA.

### Cell culture

Mouse primary hepatocytes were isolated using previously described methods[22]. Hepatocytes were cultured in DMEM containing 4.5 g/L glucose, 10% FBS, and penicillin/streptomycin. For ethanol treatment of cells, primary hepatocytes were treated with 100 mM ethanol for 48 hours. During the period of ethanol treatment, the humidity pan of the incubator was added with water containing 100 mM of ethanol to prevent ethanol volatiliaztion in cell culture medium. Both medium and humidity pan water with ethanol were replaced every 24hrs.

### Real-time RT-PCR analysis

Total RNAs were isolated from liver tissues using TRIzol reagent (Takara) according to the manufacturer's instructions and converted into cDNA using a cDNA synthesis kit (Takara). Realtime PCR analysis was performed using SYBR Green Master Mix (Takara) in ABI StepOnePlus Real-Time PCR system. The primers used in PCR reactions were as follows: mSirt1 forward, 5’-CCCTCAAGCCATGTTTGATA-3’; mSirt1 reverse, 5’-ACACAGAGACGGCTG GAACT-3’; mSirt6 forward, 5’-ACGTCAGAGACACGGTTGTG-3’; mSirt6 reverse, 5’-CCTCTA CAGGCCCGAAGTC-3’.

### Western blot analysis

Protein extracts from hepatocytes or liver tissues were made in the lysis buffer (50 mM Hepes, pH 7.5, 150 mM NaCl, 10% glycerol, 1% Triton X-100, 1.5 mM MgCl_2_, 1 mM EGTA and freshly added 1 mM PMSF and an additional protease cocktail tablet from Roche at one tablet/10 mL final buffer volume). Protein extracts were resolved on an SDS-PAGE gel and transferred to nitrocellulose membranes. Immunoblots were blocked in TBST with 5% skimmed milk for 1 hour at RT and incubated overnight at 4°C with the following primary antibodies: anti-NAMPT, anti-Actinin (Proteintech), anti-H3K9Ac, anti-H3K56Ac, anti-histone H3 (Cell Signaling Technology), anti-SIRT1 (Santa Cruz Biotechnology), anti-SIRT6 (Abcam). Following 3 washes in TBST, immunoblots were incubated with HRP-conjugated secondary antibody for 1 hour. After 3 washes in TBST, the immune complexes were detected using the ECL detection reagents (Sigma).

### NAD^+^ determination

The intracellular NAD^+^ and NADH levels were determined by a cyclic enzymatic assay. Briefly, cells (1X10^6^/reaction) or liver samples (50 mg/reaction) were lysed in acid extraction buffer (50 mM HCl, 1 mM EDTA, 300 ul for each NAD^+^ assay) or alkaline extraction buffer (50 mM NaOH, 1 mM EDTA, 300 ul for each NADH assay). All extracts were incubated at 60°C for 10 minutes to destroy endogenous enzyme activities and pyridine nucleotides. Then, the NAD^+^ and NADH extracts were neutralized by NaOH and HCl, respectively. 5 ul of each extract was then mixed with 85 ul cycling buffer (10 mM Tris, pH 8.0, 5 mM EDTA, 0.5 mM MTT, 0.2 mg/ml alcohol dehydrogenase (ADH), and 1 mM PES). The cycling reaction was initiated by adding 10 ul 6 M ethanol to each assay, and the absorbance at 570 nm was measured after 1, 3, and 6 minutes using an ELISA plate reader.

### Biochemical analysis

Hepatic and cellular lipids were extracted with chloroform/methanol (2:1), as described previously[23]. Triglyceride, cholesterol, ALT, and AST were determined using assay kits from Nanjing Jiancheng Bioengineering Institute.

### Statistical analysis

All data are presented as the mean ± S.E.M. Analysis was performed using 2-tailed unpaired Student's t-test, and *p* < 0.05 was considered as significant.

## Results

### Ethanol down-regulates *Nampt* expression in primary hepatocytes

**Fig 1:**
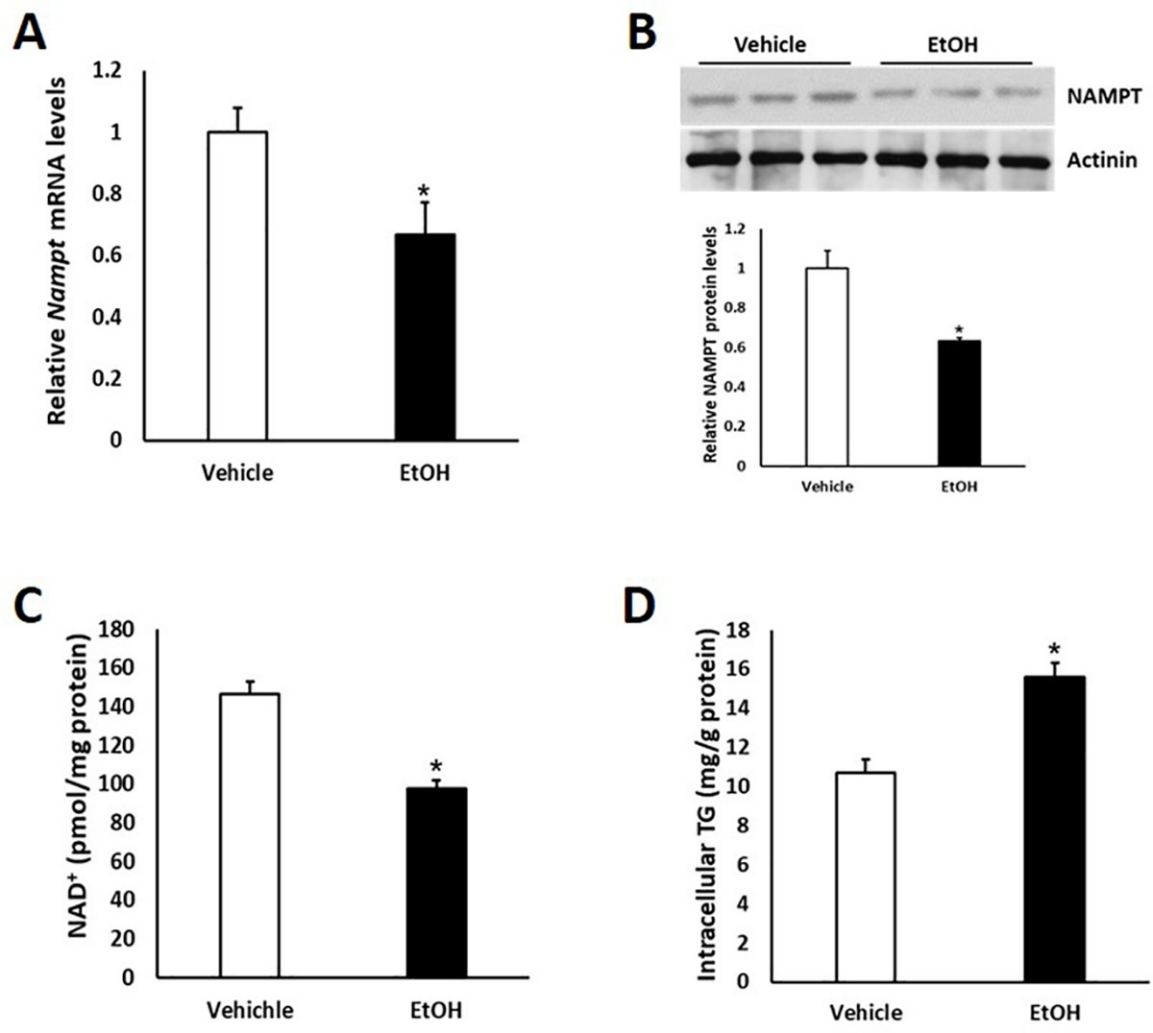
*Nampt* expression is significantly decreased in primary hepatocytes after ethanol treatment. Mouse primary hepatocytes were cultured with 100 mM ethanol for 48 hours. (A) qPCR analysis of *Nampt* expression in primary hepatocytes. (B) upper panel: western blot analysis of *Nampt* expression in primary hepatocytes; lower panel: densitometric analysis of the immunoblot data. (C and D) Intracellular NAD^+^ (C) and triglyceride (TG) (D) levels were analyzed in primary hepatocytes. Data are shown as mean ± S.E.M (n = 3 for each group). *p < 0.05 for Vehicle vs EtOH.

To explore whether ethanol treatment has an effect on *Nampt* expression in hepatocytes, we isolated mouse primary hepatocytes and treated them with 100 mM ethanol for 48 hours. Interestingly, mRNA and protein levels of *Nampt* were both markedly reduced after ethanol treatment (Fig 1A and 1B). As expected, ethanol exposure significantly increased intracellular triglyceride (TG) levels whereas NAD^+^ levels were reduced (Fig 1C and 1D).

### NAMPT overexpression increases cellular NAD^+^ contents thereby reduce triglyceride levels in primary hepatocytes treated with ethanol

**Fig 2:**
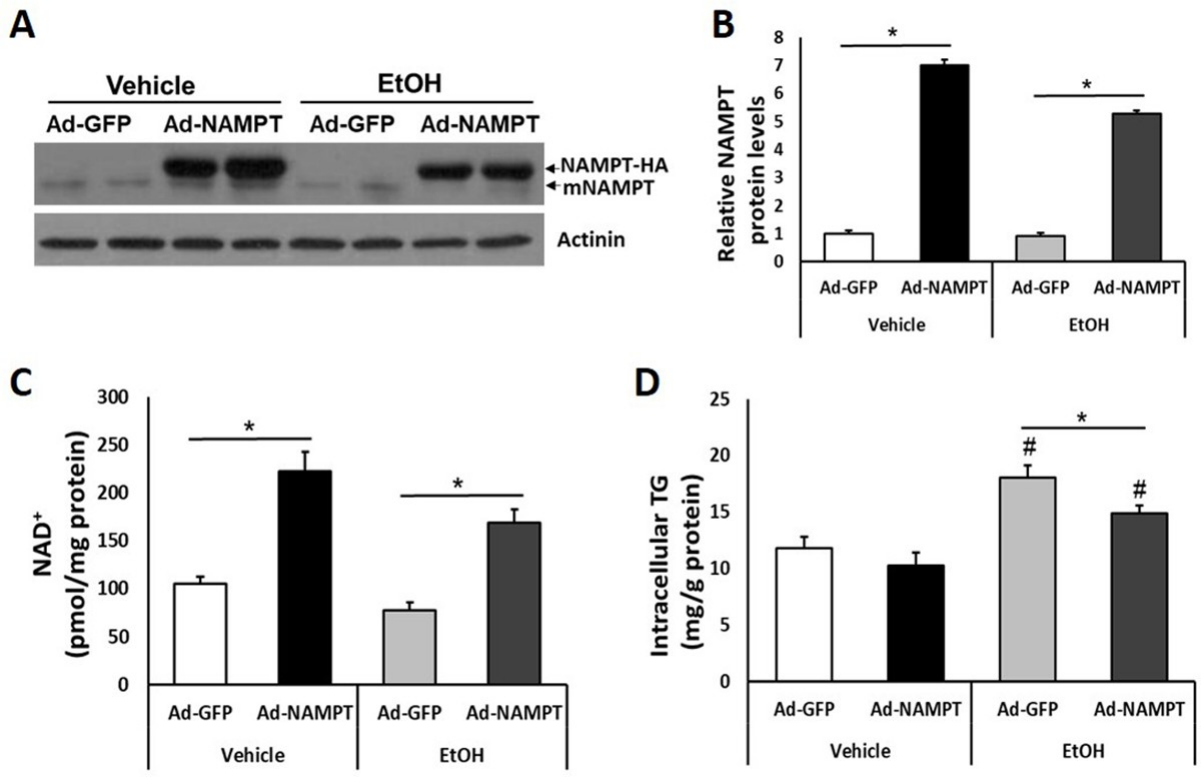
Overexpression of NAMPT elevates intracellular NAD^+^ levels and reduces TG contents in ethanol-treated primary hepatocytes. Mouse primary hepatocytes were cultured with 100 mM ethanol for 48 hours. (A) Adenovirus-mediated NAMPT-HA expression was determined by western blot. (B) Densitometric analysis of the immunoblot data in Panel A. (C and D) Intracellular NAD^+^ (C) and TG (D) levels were assessed in primary hepatocytes infected with GFP or NAMPT adenoviruses. Data are shown as mean ± S.E.M (n = 3 for each group). *p < 0.05 for Vehicle vs EtOH.

The reduced expression of *Nampt* in response to ethanol exposure in hepatocytes indicates a possible involvement of NAMPT in regulating ethanol induced intracellular TG accumulation. We next assessed the role of NAMPT in lipid metabolism in hepatocytes. In order to efficiently overexpress NAMPT in primary hepatocytes, adenovirus expressing HA-tagged human NAMPT or control adenovirus expressing GFP alone were generated. Western blot using NAMPT antibody showed significant expression of NAMPT-HA in adenovirus infected mouse primary hepatocytes (Fig 2A and 2B). 4 hours after infection with adenovirus, mouse primary hepatocytes in vehicle group and ethanol treated group were replaced culture medium and treated with saline and 100 mM ethanol for 48 hours, respectively. Overexpression of NAMPT led to an increase in intracellular NAD^+^ concentrations in both vehicle and the ethanol treated groups (Fig 2C). The elevation of NAD^+^ levels resulted in a significant reduction of intracellular TG concentrations in the hepatocytes of the ethanol treated group (Fig 2D). However, in hepatocytes of the control group, NAMPT overexpression did not significantly reduce the intracellular TG levels remarkably (Fig 2D).

### Chronic ethanol administration induces hepatic steatosis and reduces *Nampt* expression in mouse liver

**Fig 3:**
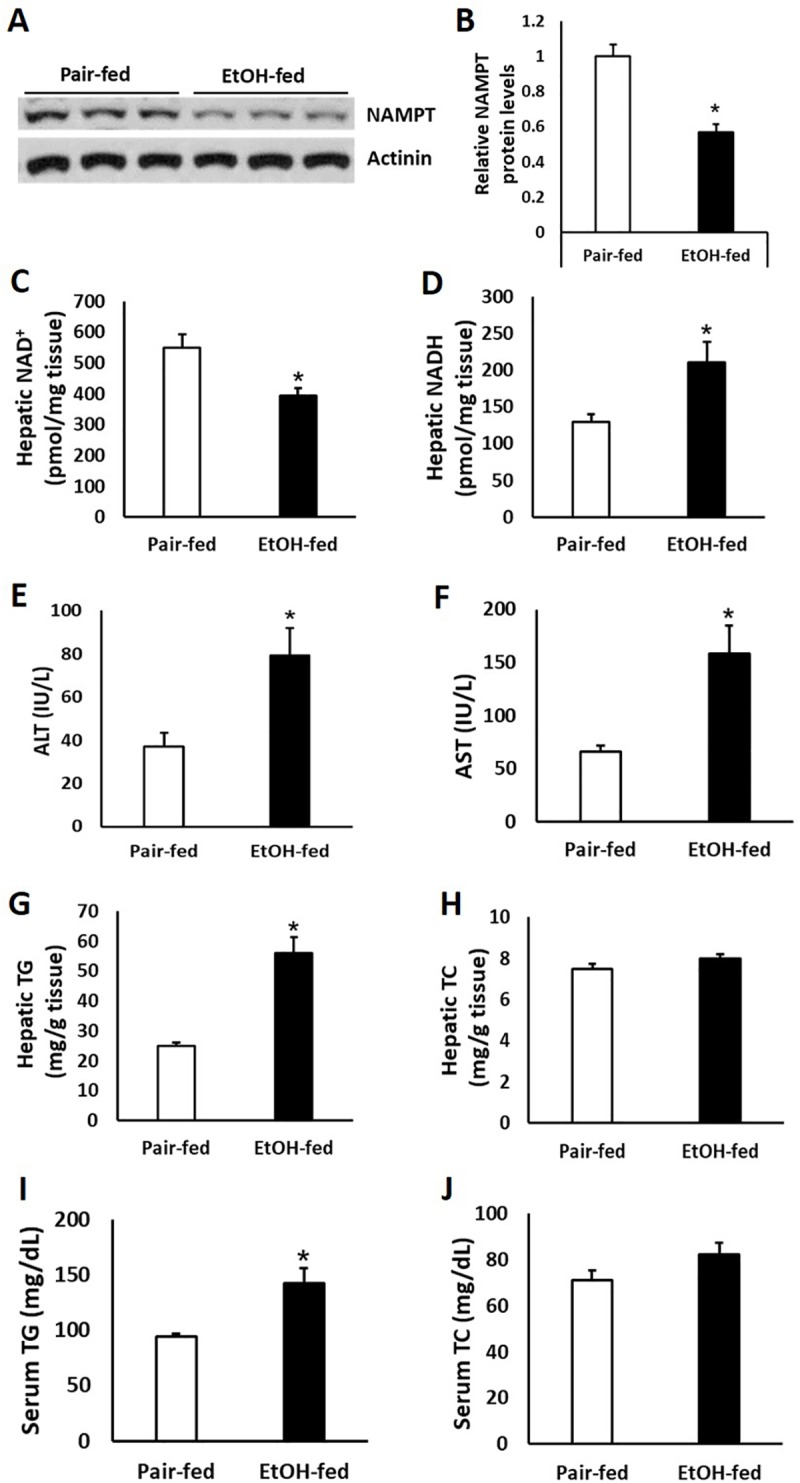
Chronic-binge ethanol feeding induces hepatic steatosis and reduces *Nampt* expression in mouse livers. C57BL/6J mice were fed control or ethanol diet for 10 days, followed by single gavage of maltose or ethanol, respectively. Mice were euthanized 9 hours after gavage. (A) Western blot analysis of *Nampt* expression in mouse livers. (B) Densitometric analysis of the immunoblot data in Panel A. (C and D) NAD^+^ (C) and NADH (D) concentrations were measured in mouse livers. (E and F) Serum ALT (E) and AST (F) levels were examined. (G-J) Hepatic TG, total cholesterol (TC) (G, H); serum TG, TC (I, J) levels were assessed. Data are shown as mean ± S.E.M (n = 5–6 for each group). *p < 0.05 for Pair-fed vs EtOH-fed.

To test our hypothesis that ethanol may reduce *Nampt* expression *in vivo*, we performed western blot to evaluate NAMPT protein levels in liver tissue of a mouse model of chronic plus binge ethanol feeding[21]. As expected, we found that NAMPT protein was more abundant in pair-fed than in ethanol-fed mouse livers (Fig 3A and 3B). Since ethanol metabolism in hepatocytes reduces available NAD^+^ while increases NADH, ethanol administration reduced NAD^+^ concentrations while upregulated NADH levels in mouse livers (Fig 3C and 3D). As has been reported, ethanol feeding induced significantly higher levels of serum ALT and AST (Fig 3E and 3F). Hepatic and serum levels of TG were much higher in the ethanol-fed group compare to the pair-fed group, whereas liver and serum levels of cholesterol (TC) were comparable between these two groups (Fig 3G–3J).

### NAMPT overexpression alleviates ethanol-induced liver steatosis and injury

**Fig 4:**
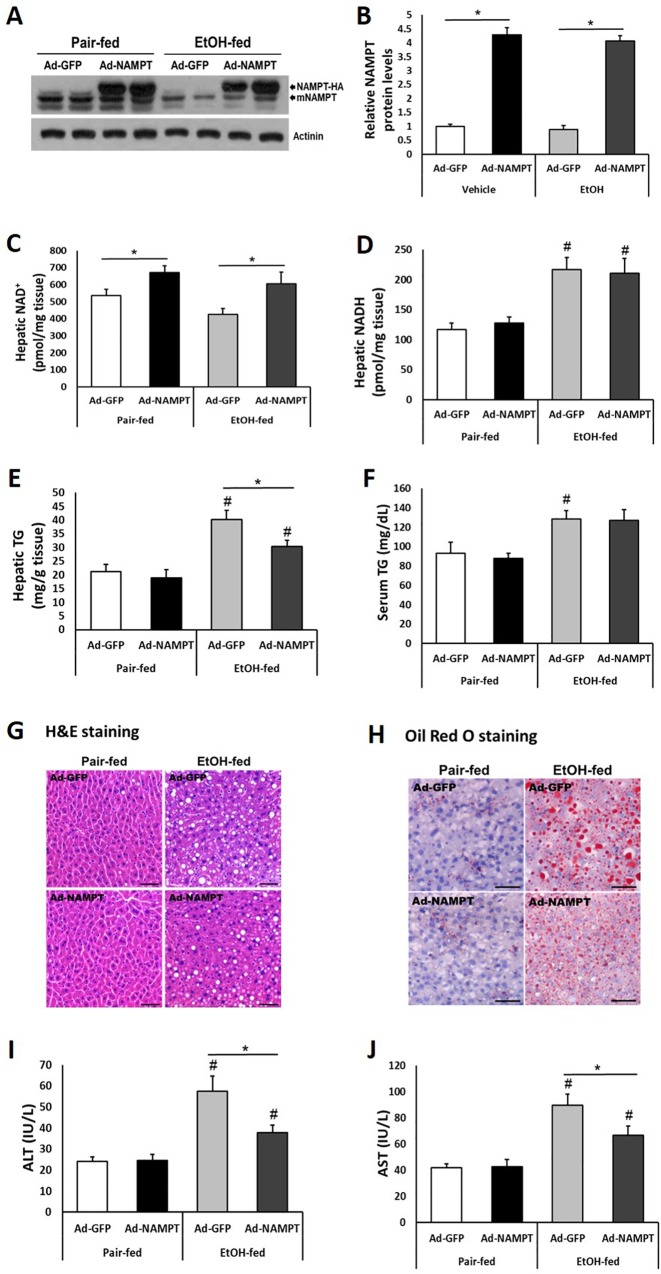
NAMPT overexpression ameliorates chronic-binge ethanol-induced fatty liver injury in mice. Mice were fed with control or ethanol diet as described in Fig 3; adenoviruses were injected into mice through tail vein at a dosage of 1x10^9^ pfu/mouse. (A) Adenovirus-mediated NAMPT-HA expression was analyzed by western blot. (B) Densitometric analysis of the immunoblot data in Panel A. (C and D) NAD^+^ (C) and NADH (D) concentrations were examined in mouse liver tissues. (E and F) Hepatic and serum TG levels were assessed. (G and H) H&E staining (G) and oil-red O staining (H) of liver sections. Scale bar, 50 μm. (I and J) Serum ALT (I), AST (J) levels were analyzed. Data are shown as mean ± S.E.M (n = 5–6 for each group). *p < 0.05 for Ad-GFP vs Ad-NAMPT; #*p* < 0.05 vs Pair-fed Ad-GFP.

We took advantage of the relative tissue specificity of adenovirus for liver to create a relatively liver-specific overexpression of NAMPT[24]. We delivered NAMPT and control GFP adenovirus to pair-fed and ethanol-fed mice via tail vein injections. Adenovirus-mediated NAMPT-HA expression in liver was about 4–5 times higher than endogenous NAMPT expression (Fig 4A and 4B). As a result, hepatic NAD^+^ levels were increased by ~25% and 42% in NAMPT overexpressed pair-fed and ethanol-fed mouse livers, respectively (Fig 4C). Indeed, hepatic NADH levels were increased by ~75% in mice after ethanol feeding (Fig 4D). However, hepatic NADH levels were not regulated by NAMPT in neither pair-fed nor ethanol-fed mice (Fig 4D). As expected, NAMPT overexpression in liver significantly reduced hepatic TG concentrations and serum ALT, AST levels in ethanol-fed mice but not in pair-fed mice (Fig 4E and 4G–4J). However, NAMPT overexpression had no effect on serum TG levels in both pair-fed and ethanol-fed mice (Fig 4F).

### Chronic ethanol feeding reduces the expression of *Sirt1* and *Sirt6* in liver

**Fig 5:**
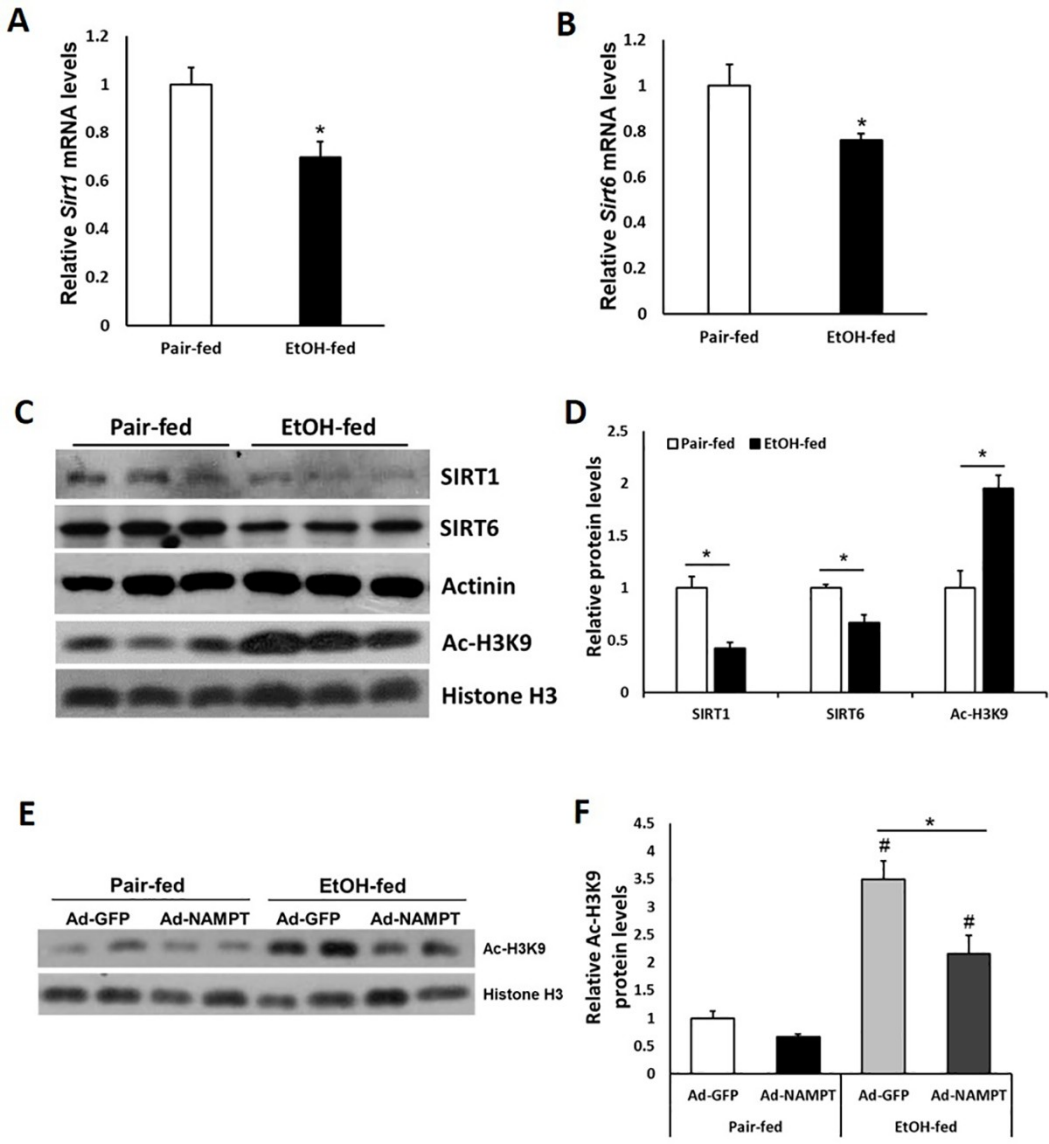
Chronic-binge ethanol feeding suppresses the expression of *Sirt1* and *Sirt6* in mouse livers. Mice were fed with control or ethanol diet as described in Fig 3. (A and B) Relative hepatic mRNA levels of *Sirt1* and *Sirt6* were analyzed by qPCR. (C) Western blot analysis of hepatic SIRT1, SIRT6 and Ac-H3K9 protein levels were examined. (D) Densitometric analysis of the immunoblot data in Panel C. (E and F) Western blot analysis (E) and densitometric analysis (F) of Ac-H3K9 in liver tissues of Ad-NAMPT and Ad-GFP adenoviruses treated mice fed with control or ethanol diet. Data are shown as mean ± S.E.M (n = 4 per group). *p < 0.05 for Pair-fed vs EtOH-fed; #*p* < 0.05 vs Pair-fed Ad-GFP.

Sirtuins belong to a family of NAD^+^-dependent protein deacetylase. Sirtuin activities are regulated by cellular NAD^+^ levels and therefore influenced by the NAD^+^/NADH ratios[25]. Because SIRT1 and SIRT6 have been shown to play roles in hepatic lipid metabolism, we next analyzed the expression of *Sirt1* and *Sirt6* in the livers of mice exposed to ethanol. As expected, mRNA and protein levels of both SIRT1 and SIRT6 were significantly decreased in the livers of ethanol-fed mice compared with pair-fed mice (Fig 5A–5D). Since both SIRT1 and SIRT6 function as a histone deacetylase on lysine 9 of histone H3[25], reduction of SIRT1/SIRT6 also led to increased acetylation on the site of histone H3K9 in ethanol fed mouse livers (Fig 5C and 5D). Furthermore, NAMPT overexpression remarkably reduced the acetylation levels of histone H3K9 site in ethanol-fed mouse livers probably through upregulating sirtuin activities (Fig 5E and 5F).

### SIRT1 mediates the effects of NAMPT on ethanol-induced hepatic steatosis and injury

**Fig 6:**
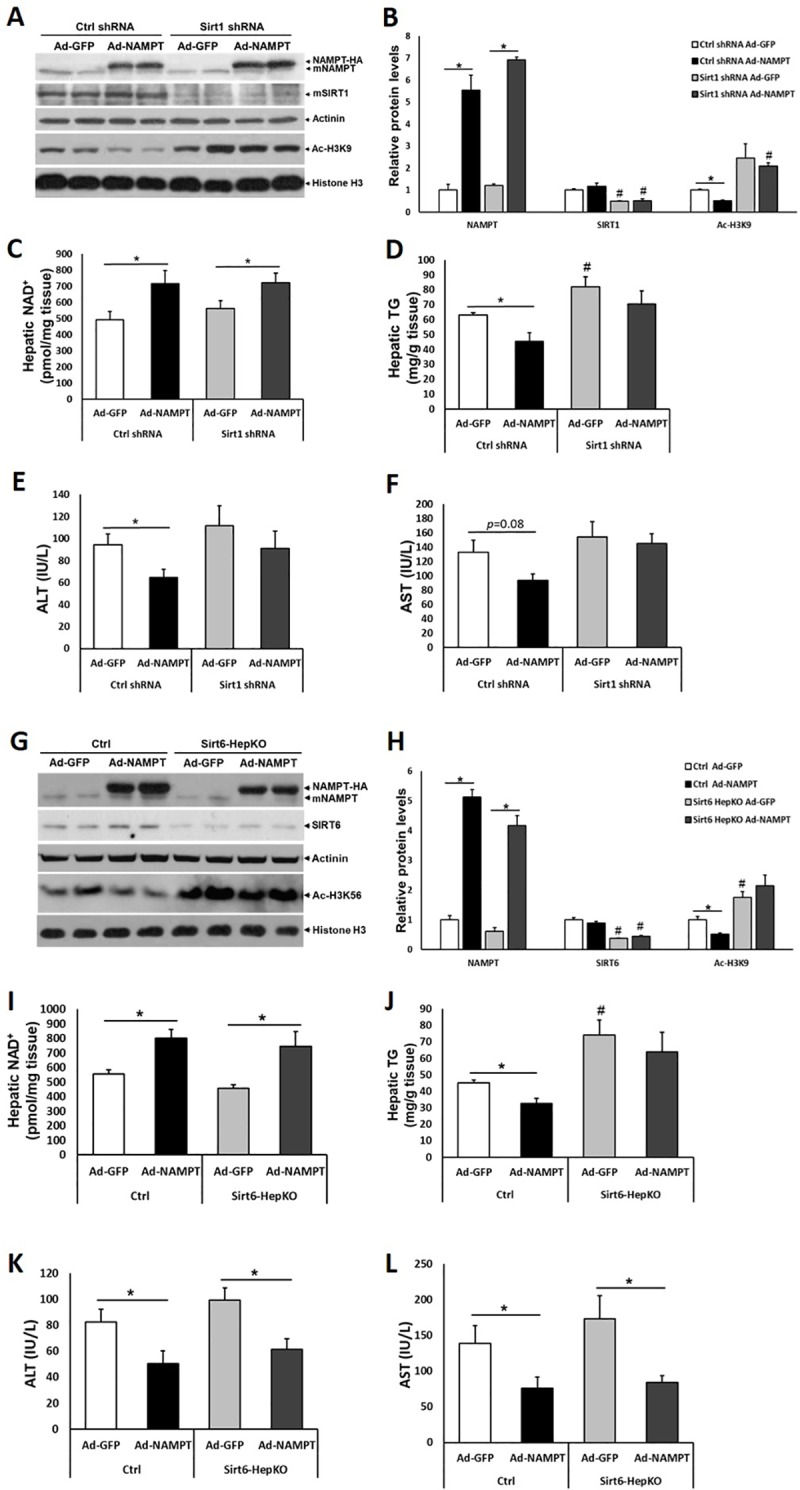
SIRT1 is required for the beneficial effects of NAMPT on ethanol-induced hepatic steatosis and injury. Mice were fed with ethanol diet as described in Fig 3; adenovirus treatments were performed as in Fig 4. (A) Adenovirus-mediated *Sirt1* shRNA and NAMPT-HA expression as well as histone H3K9 acetylation levels were examined by western blot. (B) Densitometric analysis of the immunoblot data in Panel A. (C and D) NAD^+^ (C) and TG (D) concentrations were examined in mouse liver tissues. (E and F) Serum ALT (E), AST (F) levels were analyzed. (G) Adenovirus-mediated NAMPT-HA expression as well as protein levels of SIRT6 and Ac-H3K56 were examined by western blot. (H) Densitometric analysis of the immunoblot data in Panel G. (I and J) NAD^+^ (I) and TG (J) concentrations were examined in mouse liver tissues. (K and L) Serum ALT (K), AST (L) levels were analyzed. Data are shown as mean ± S.E.M (n = 5–6 for each group). *p < 0.05 for Ad-GFP vs Ad-NAMPT; #*p* < 0.05 vs Ctrl shRNA Ad-GFP in Panel B and D; #*p* < 0.05 vs Ctrl Ad-GFP in Panel H and J.

Since SIRT1 has been shown to regulate ethanol-induced liver steatosis[26], we hypothesized that increased NAD^+^ production and SIRT1 activity caused by NAMPT overexpression contributed to the alleviation of ethanol-induced hepatic steatosis. To test our hypothesis, we used a combination of adenoviruses to simultaneously overexpress NAMPT and knockdown SIRT1 in the livers of mice fed with ethanol. Adenovirus treatments successfully overexpressed NAMPT and lowered *Sirt1* expression in mouse livers (Fig 6A and 6B). Since SIRT1 has been reported to deacetylate histone H3 lysine 9 (H3K9)[25]. As expected, the acetylation level of histone H3K9 was significantly upregulated after *Sirt1* knockdown (Fig 6A and 6B). Previous studies have shown that specific ablation of SIRT1 in liver is associated with rapid onset and progression of steatosis in response to ethanol exposure[27]. Consistently, in our study, SIRT1 knockdown led to increased hepatic TG accumulation in mice fed with ethanol (Fig 6D). Importantly, NAMPT overexpression led to elevation of liver NAD^+^ levels, which is SIRT1 independent (Fig 6C). As expected, overexpression of hepatic NAMPT led to reduced TG concentrations and serum ALT, AST levels, but this effect was abolished when SIRT1 was knocked down, suggesting SIRT1 mediates the effects of NAMPT on ethanol-induced liver steatosis and injury (Fig 6D–6F).

Moreover, SIRT6 has also been implicated in the regulation of lipid metabolism in liver[25, 28]. However, the role of SIRT6 in ALD remains unclear. We generated a *Sirt6* hepatocyte-specific knockout mouse model (Sirt6-HepKO) using floxed *Sirt6* mouse strain and an Albumin-Cre line. To test whether SIRT6 mediates the effects of NAMPT on ethanol-induced liver steatosis, we also used adenovirus to overexpress NAMPT-HA in the livers of Sirt6-HepKO mice fed with ethanol. NAMPT-HA expression in liver was 4–5 times higher than endogenous NAMPT (Fig 6G and 6H). Indeed, a SIRT6 specific substrate, acetylated histone H3 lysine 56 (Ac-H3K56), was increased in the liver due to *Sirt6* ablation (Fig 6G and 6H). NAMPT overexpression elevated NAD^+^ levels in both control and Sirt6-HepKO groups (Fig 6I). Interestingly, after ethanol feeding, hepatic TG levels were elevated in the Sirt6-HepKO mice compared to the control mice (Fig 6J). NAMPT overexpression reduced hepatic TG levels in ethanol-fed control mice but not in Sirt6-HepKO mice, indicating SIRT6 is also required for NAMPT’s effects on alleviating hepatic steatosis (Fig 6J). However, overexpression of NAMPT led to reduced serum ALT and AST levels in both ethanol-fed control and Sirt6-HepKO mice, suggesting SIRT6 is not critical to mediate the effects of NAMPT on lowering serum levels of ALT and AST (Fig 6K and 6L). Taken together, our data suggest that SIRT1 is the major sirtuin family member to mediate NAMPT’s role in alleviating ethanol-induced liver steatosis and injury.

## Discussion

Disturbances in NAMPT and NAMPT-mediated NAD^+^ biosynthesis have been reported to contribute to the development of non-alcoholic fatty liver disease (NAFLD) and NAMPT is implicated in the regulation of hepatic lipid metabolism[14–16, 19]. However, whether NAMPT modulates the processes involved in the pathogenesis of alcoholic liver disease (ALD) still remains unknown. In this study, we showed that adenovirus-mediated hepatic overexpression of NAMPT increased NAD^+^ levels and thereby alleviated ethanol-induced liver steatosis. Mechanistically, we demonstrated that SIRT1 was required to mediate the effects of NAMPT on lowering of liver TG accumulation and serum ALT, AST levels.

Hepatic NAD^+^ content or NAMPT abundance is known to decrease in HFD-fed animals[14–16]. NAFLD and ALD are both steatohepatitic processes and share many common features[20], however, the relationship between NAMPT abundance and ALD development is still unknown. Since ethanol metabolism in liver reduces NAD^+^ availability, we firstly postulated that *Nampt* expression in liver might be adaptively increased to produce more NAD^+^, thereby to meet the requirements of ethanol oxidation. Surprisingly, we found that hepatic *Nampt* expression was significantly reduced after ethanol treatment *in vitro* and *in vivo*. Although the importance of NAMPT in the regulation of NAD^+^ homeostasis and metabolism has been extensively studied, it is not well understood how this gene is regulated. Interestingly, the *Nampt* gene has been found to be a circadian clock-controlled gene[29, 30]. Thus, the impairment of the hepatic circadian clock activity caused by ethanol treatment may contribute to the reduction of *Nampt* expression in ethanol-fed mouse livers[31]. In addition, a recent study has shown that hepatic *Nampt* expression is regulated by FoxO transcription factors[18]. Ethanol administration increases the acetylation of FOXO1 through inhibition of SIRT1 activity, and subsequently downregulates the transcriptional activity of FOXO1. Thus, the reduced expression of *Nampt* may be caused by ethanol’s inhibition of FOXO1 transcriptional activity[32]. Taken together, the reduced NAMPT-driven NAD^+^ salvage biosynthesis, together with the increased level of NAD^+^ consuming biological processes (e.g. ethanol oxidation), results in an overall depletion of the hepatic NAD^+^ pool and thus leads to the development of ALD.

Supplementation with NAD^+^ precursors or inhibition of NAD^+^ consuming enzymes leads to an increase of NAD^+^ levels thereby protecting against metabolic disorders in animals[15, 33–35]. The metabolism of ethanol in liver increases the conversion of NAD^+^ to NADH, resulting in a reduction of the ratio of NAD^+^/NADH[2]. Several studies have demonstrated that elevation of hepatic NAD^+^ content through NA or NR supplementation or pharmacological inhibition of PARP attenuates ethanol-induced hepatic steatosis[36–38]. In this study, we showed for the first time that NAMPT overexpression is an alternative way to enhance NAD^+^ biosynthesis and alleviate ethanol-induced fatty liver.

Among the NAD^+^‐dependent sirtuin family members, SIRT1 is the best characterized regulator in metabolism, inflammation and ageing[39]. Rodent and human studies show that ethanol-mediated disruption of the hepatic SIRT1 signaling plays a critical role in the development of ALD[26]. Suppression of SIRT1 by ethanol inhibits or stimulates the activities of many transcriptional factors and co-regulators, including AMPK, SREBP-1, PGC-1ɑ and Lipin-1, thereby deregulating diverse metabolism and inflammation related pathways including *de novo* lipogenesis, fatty acid β-oxidation, lipoprotein uptake and secretion, and production of pro-inflammatory cytokines in the liver[26, 27, 40–43]. Although we have not directly assessed the SIRT1 activity in our study, increased acetylation of histone H3K9 site in ethanol-fed mouse livers indicates a reduction of SIRT1 activity caused by ethanol treatment. Moreover, hepatic *Sirt1* mRNA and protein expression remarkably decreased in mice after ethanol feeding. These evidences prompted us to study whether SIRT1 mediated the effects of NAMPT on ethanol-induced liver steatosis. Indeed, our results indicate that SIRT1 is an important mediator for these beneficial effects of NAMPT. As shown in our study, knockdown of SIRT1 abolished (but not completely) NAMPT effects on lowering liver TG concentrations and serum ALT, AST levels. Thus, we still believe that other NAD^+^-sensitive factors may contribute to the beneficial effects of NAMPT. For example, we also observed a reduction of *Sirt6* expression in the livers of ethanol-fed mice. Therefore, Sirt6-HepKO mice were used to evaluate whether SIRT6 is also required for NAMPT’s beneficial effects. Interestingly, NAMPT overexpression did not lower hepatic TG levels in Sirt6-HepKO mice. In contrast, serum ALT and AST levels were reduced by NAMPT overexpression in both control and Sirt6-HepKO mice. Therefore, these data suggest that SIRT6 is required to regulate hepatic steatosis but not liver injury downstream of NAMPT. Taken together, SIRT1 is the most important sirtuin family members lies downstream of NAMPT to regulate hepatic steatosis and injury.

In summary, the present study demonstrates that NAMPT-mediated NAD^+^ biosynthesis is severely compromised in liver by ethanol feeding. Thus, hepatic overexpression of NAMPT restores NAD^+^ levels, and thereby alleviates ethanol-induced hepatic steatosis in a SIRT1-dependent manner. Taken together, our data indicate that activation of NAMPT might be an effective way to prevent adverse effects induced by alcohol consumption.

## Supporting information

S1 File The raw data for each figure. (XLSX)
